# Supplementary material for: Real-time monitoring of oncolytic VSV properties in a novel in vitro microphysiological system containing 3D multicellular tumor spheroids
Source: PLoS One. 2020 Jul 6;15(7):e0235356. doi: 10.1371/journal.pone.0235356 (PMC7337297; doi:10.1371/journal.pone.0235356)
Supplement: Attachment — Submitted filename: Reviewer comments.docx [file pone.0235356.s001.docx]

**Reviewer’s comments**

Authors have investigated the potential of their recently developed microphysiological system (MPS) that mimics the *in vivo* tumor microenvironment and serves as culture space for 3D multicellular tumor spheroids (MCTS). This system was used as a model to check the oncolytic potential of Vesicular Stomatitis virus and concluded that this system can be used as a first line of monitoring system to validate any oncolytic virus efficacy. It is a helpful model system for evaluation of anticancer therapies including virotherapy.

It is a good piece of work; however it was not presented very well. Methodology and results need to be written clearly. At many places the clarity is missing. Experiment description in the legends needs lot of improvement.

**Comment 1:** In Figure 1C, A549 cells stained green and MRC5 red. But why is it bright yellow at the interface of these two cell populations? Is it due to overlap?

**Comment 2**: Reference to the “**effective replication of oncolytic VSV expressing GFP in A549cells**” under result section, related Fig 2C, oVSV effects were shown at 5h and 50h. It would be better, if 25h data of GFP expression and PI staining is also incorporated in the image panel. In this experiment, CPE/GFP expression/cell death was measured up to 50h post infection; but the negative control (mock infected) was not included. How was it confirmed without a negative control that the noted cell death was virus induced?

**Comment 3**: In Figure 3E, quantification of viral genome copies was done to analyse oVSV-GFP replication in MCTS eluates. How was it done? Was it the amplification of VSV –G gene by quantitative PCR? Please clarify in the text and legend. In this experiment as well, negative control was missing.

**Comment4**: What was the rationale behind using different MOIs, 1 and 5 for infections in 2D cultures and MPS?

**Comment 5:** From the methodology section given under ‘**quantification of secreted human interferon beta (IFNβ) after VSV-GFP infection**’ it is clear that IFNβ response was measured in all three types of cells following oVSV-GFP infection in 2D cultures and eluates collected from infected MPS by ELISA. But the related result and Figure/ figure legend are confusing. Please check the result and the figure related to this experiment and write more in detail.

**Comment 6:** To check the differential replication of oVSV-GFP in the 3D MCTS integrated MPS, A549cells, MRC-5 cells were labeled with fluorescence tracker. Figure 4A and 4B, both the cells; A549 and MRC5 were stained red. How were these two cell populations separated to check the expression oVSV-GFP through red and green merge? Are 4A and 4B images from the same spheroid? Clarify and also mention in the text and legends. In the figure 1, A549 cells were stained green.

**Comment 7:** There is a lot of room for improvement in discussion section. One sentence in the discussion from lines 326 to 329 is the exact repetition of a sentence under result section from lines 281 to 284. Improve the discussion.

**Comment 8**: The intensity of GFP expression in A549cells does indicate the active replication of oVSV-GFP virus but not the anti tumor activity. Experiment of Live/Dead cell staining following virus infection does not commend the anti tumor activity of oncolytic virus used. An anti tumor/ anti proliferative marker may be used to confirm the anti tumor activity of the virus in MCTSs. Following infection, spheroids can be checked for the expression of aforementioned markers.
